# Central autonomic network alterations in male endurance athletes

**DOI:** 10.1038/s41598-022-20064-3

**Published:** 2022-10-06

**Authors:** Feliberto de la Cruz, Maria Geisler, Andy Schumann, Marco Herbsleb, Zora Kikinis, Thomas Weiss, Karl-Jürgen Bär

**Affiliations:** 1 Lab for Autonomic Neuroscience, Imaging and Cognition (LANIC), Department of Psychosomatic Medicine and Psychotherapy, Jena University Hospital, 07743 Jena, Germany; 2 Department of Clinical Psychology, Friedrich-Schiller-University Jena, 07743 Jena, Germany; 3 Psychiatry Neuroimaging Laboratory, Department of Psychiatry, Brigham and Women’s Hospital, and Harvard Medical School, Boston, MA 02115 USA

**Keywords:** Magnetic resonance imaging, Neuroscience

## Abstract

Physical exercise causes marked adjustments in brain function and the cardiovascular system. Brain regions of the so-called central autonomic network (CAN) are likely to show exercise-related alterations due to their involvement in cardiac control, yet exercise-induced CAN changes remain unclear. Here we investigate the effects of intensive exercise on brain regions involved in cardiac autonomic regulation using resting-state functional connectivity (rsFC). We explored rsFC of six core regions within CAN, namely ventromedial prefrontal cortex, dorsolateral anterior cingulate cortex, left/right amygdala, and left/right anterior insula, in 20 endurance athletes and 21 non-athletes. We showed that athletes had enhanced rsFC within CAN and sensorimotor areas compared to non-athletes. Likewise, we identified two networks with increased rsFC encompassing autonomic and motor-related areas using network-based statistics analysis. In addition, rsFC displayed an inverse relationship with heart rate, where the stronger rsFC in athletes correlates with their slower heart rate. Despite this significant relationship, mediation analysis revealed that heart rate is a weak mediator of the effect of intensive physical training on rsFC. Our findings prove that physical exercise enhances brain connectivity in central autonomic and sensorimotor networks and highlight the close link between brain and heart.

## Introduction

Physical activity is beneficial for our health and well-being. Regular physical exercise influences the functioning of various body organs and ultimately translates into a reduced all-cause mortality risk^1^. It has been accepted that the brain and the heart might benefit most from physical activity. Evidence suggests that sports reinforce neural networks and induce plastic changes due to the acquisition and execution of motor skills during training^2^, while causing a specific combination of structural and cardiovascular changes with improved cardiac autonomic regulation at the heart level^3^. In this vein, a large body of research emphasizes the autonomic nervous system to play a major role in mediating the adaptive processes^4,5^. Regular exercise induces a shift of the autonomic balance towards parasympathetic predominance with a concurrent decrease in sympathetic modulation. As a result, athletes and physically very active individuals tend to have slower heart rates and higher heart rate variability (HRV) in comparison to the overall healthy population. These physiological adaptations are thought to be a sign of an efficient and health-promoting cardiovascular regulation^6^.

Recent studies on brain and heart function move away from the traditional view of single-organ research and instead commence to pay close attention to what is referred to as the brain-heart axis^7–11^. Such studies rely on the basic assumption that a bi-directional flow of information exists between the brain and heart, allowing both organs to influence one another ensuring body homeostasis. In this direction, novel methodological approaches such as time-delay analysis^12^, generative models^13^, Granger Causality^14^, functional connectivity analysis^15^ or multifractal^16^ have advanced the understanding of the brain-heart axis. Although most studies have focused on descending neural signals influencing the heart^17,18^, research into ascending signals has recently gained more attention, particularly from the perspective of neuroimaging studies^19,20^. It is well-accepted that peripheral signals, such as heart rate, modulate brain dynamics at rest and play a crucial role in shaping the activity of resting-state networks^11,21^. The ascending path begins at the heart, where fluctuations of the cardiac cycle are transformed into neural signals by mechanoreceptors located in the heart and aortic wall^22^, reaching numerous cortical and subcortical structures including the insula, the amygdala, the ventral anterior cingulate cortex (vACC) and the somatosensory cortex. Thus, several studies have highlighted the involvement of ascending cardiac signals in cognitive and perceptual processes, such as self-consciousness, conscious visual experience, or memory^23,24^. On the other hand, modulation of the heart’s activity occurs through the parasympathetic and sympathetic branches of the autonomic nervous system. This top-down communication is orchestrated through the so-called central autonomic network (CAN^25^), a subgroup of regions mainly including cortico-limbic and brainstem structures. The most prominent CAN regions are the ventromedial prefrontal cortex (vmPFC), dorsolateral anterior cingulate cortex (dACC), anterior insula (aINS) and the amygdala, which all form an interconnected network and modulate the activity of downstream regions. While the specific autonomic function of all these brain regions is not entirely understood, most authors agree that vmPFC and dACC are engaged in sympathetic regulation. In contrast, aINS and amygdala seem to be involved in both parasympathetic (vagal) and sympathetic control^26^.

The advent of functional magnetic resonance imaging (fMRI) has made it possible to track adaptive brain functional changes in response to regular physical training^2^. Sie and colleagues^27^ used rs-fMRI to examine the effect of several levels of exercise experience in baseball players on CAN connectivity. They showed that athletes had enhanced connectivity within and between CAN regions and sensorimotor network areas depending on the level of exercise experience, providing evidence that increasing levels of sporting experience can enhance intrinsic functional connectivity of CAN areas differently. To our best knowledge, this was the only study exploring the effect of physical activity on CAN connectivity; however, it did not evaluate how physical training may affect the brain-heart axis. Of note, physical and physiological characteristics of athletes vary across sport types^28^. For example, in endurance sports, which requires practice over long distances for prolonged periods like running, swimming or cycling, athletes might develop a different physiological profile than baseball players. Evidence suggests that longer-duration exercise increases oxidative capacity and impairs cognitive control and prefrontal cortex oxygenation^29,30^, while skilled baseball players have enhanced cognitive control, inhibitory functions, and visual skills^31^. Thus, to what extent CAN connectivity changes occur in other sport types remain unclear.

The present study aims to explore the effects of regular physical exercise on CAN connectivity and the brain-heart axis in endurance athletes. Based on the previous findings of Sie and colleagues^27^, we hypothesized that endurance athletes have enhanced connectivity between CAN regions in comparison to non-athletes. To test this, we compared rsFC of core regions of the CAN, namely vmPFC, dACC, aINS and amygdala, between athletes and non-athletes. To yield a broader sense of how the whole-brain network might change in endurance athletes, we conducted a network-based statistics (NBS) analysis. NBS is a novel graph approach which does not require an apriori seed region to detect rsFC differences between groups^32^. To examine the influence of high-intensity training on spontaneous regional brain activity and to explore whether regional changes in brain activity may explain rsFC differences between athletes and non-athletes, we computed the (fractional) amplitude of low-frequency fluctuations (f)ALFF. ALFF is another well-validated data-driven technique used as an alternative to seed-based analysis to quantify spontaneous brain activity at the local level^33^. Finally, since regular physical exercise simultaneously affects heart rate and rsFC and the fact that heart rate can influence rsFC, we hypothesized that heart rate mediates the effect of regular physical exercise on CAN connectivity.

## Results

### Functional connectivity analyses

**Figure 1:**
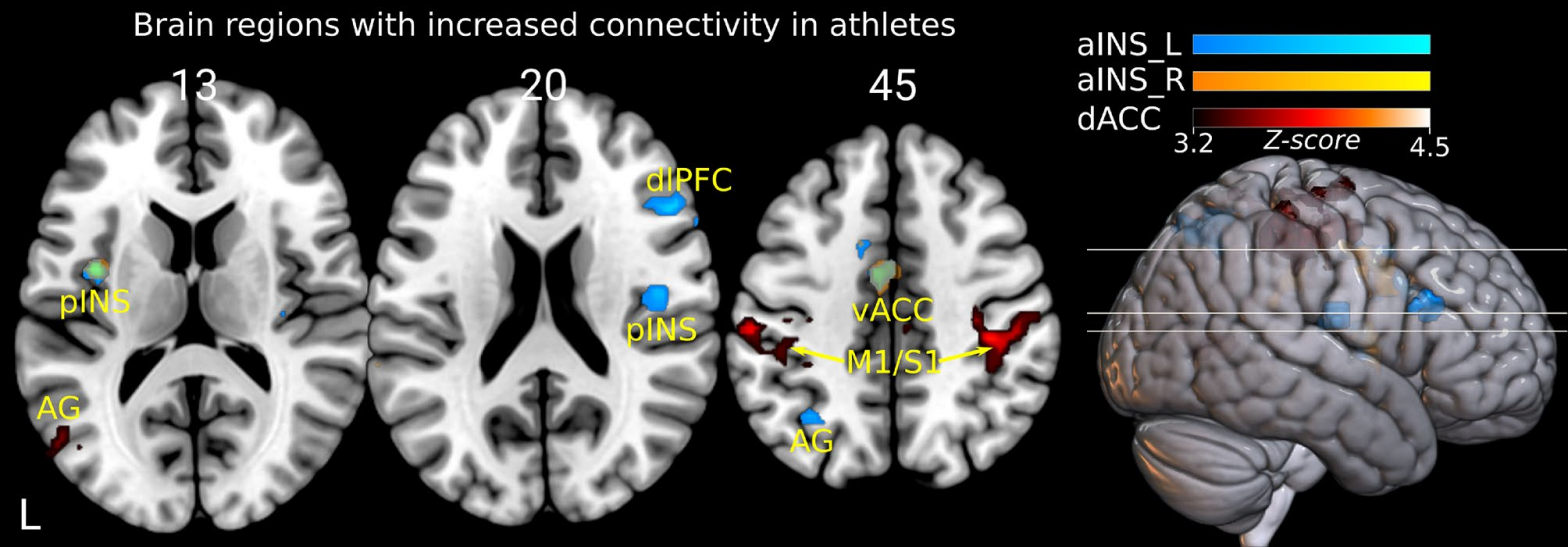
Group differences between athletes and non-athletes in functional connectivity for aINS_L, aINS_R, and dACC seeds. All identified brain regions indicate significantly increased rsFC in athletes compared to non-athletes. The cool, warm and red colormaps illustrate the magnitude of the statistical significance (Z-score) in each voxel, where brighter color means higher Z-score. The light green color found in pINS and vACC regions occurs due to the overlapping in these areas of the cool and warm colormaps from aINS_L and aINS_R, respectively. aINS_L—left anterior insula, aINS_R—right anterior insula, dACC—dorsal anterior cingulate cortex, pINS—posterior insula, AG—angular gyrus, dlPFC—dorsolateral prefrontal cortex, vACC—ventral anterior cingulate cortex, M1/S1—primary sensorimotor cortex.

**Figure 2:**
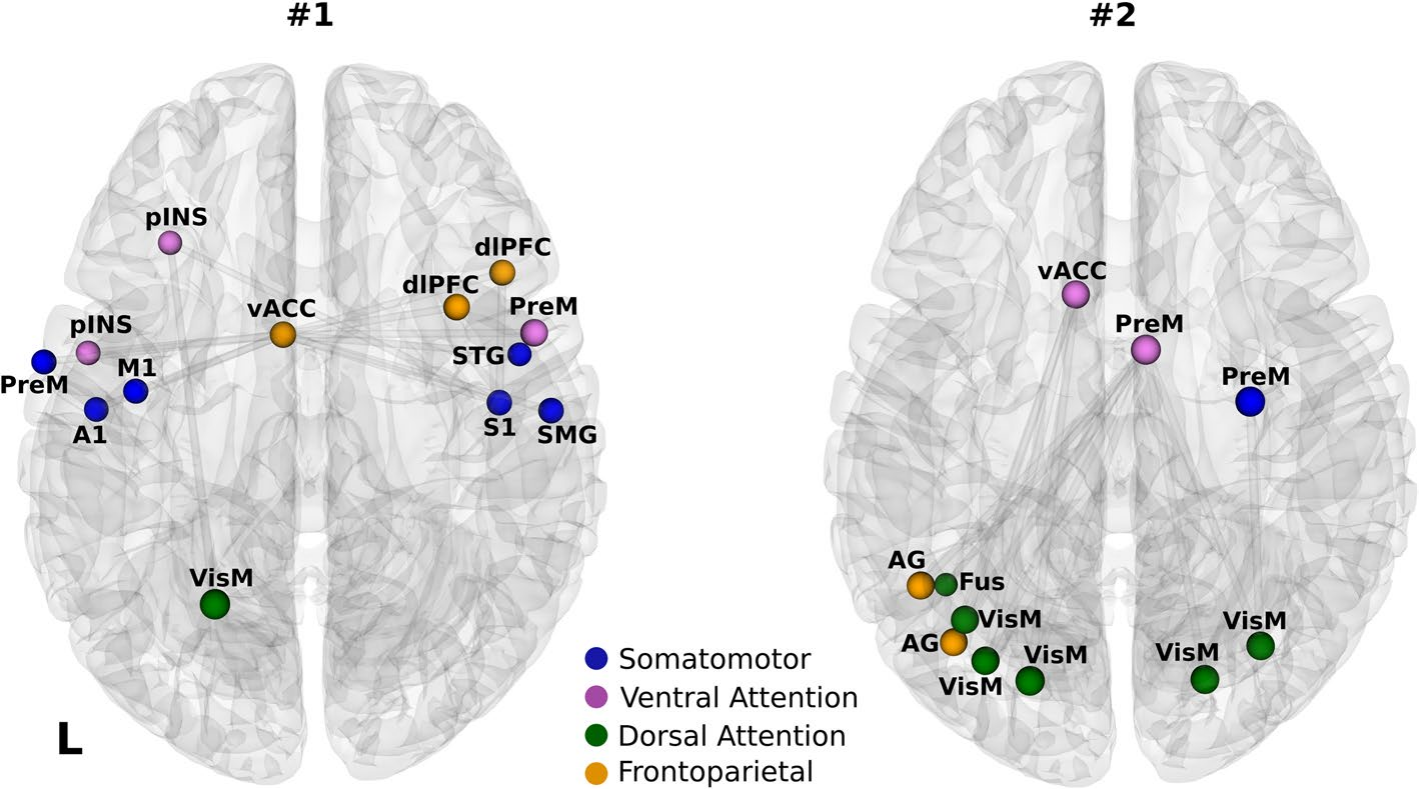
Athletes showed higher rsFC than non-athletes in two distinct network components. Network component #1 consisted of 13 nodes and 13 edges, and network component #2 consisted of 11 nodes and 13 edges, and were distributed across the somatomotor, dorsal/ventral attention and frontoparietal networks. Nodes are color-coded according to the 7 Yeo network parcellation ^54^. Somatomotor (blue), ventral attention (magenta), dorsal attention (orange) and frontoparietal (pink) networks. pINS—posterior insula, dlPFC—dorsolateral prefrontal cortex, vACC—ventral anterior cingulate cortex, M1—primary motor cortex, PreM—premotor cortex, VisM—visuomotor cortex, S1—primary sensory cortex, A1—primary auditory cortex, SMG—supramarginal gyrus, STG—superior temporal gyrus, AG—angular gyrus, Fus—fusiform gyrus.

Among the six seeds CAN regions, we found significant differences in rsFC between athletes and non-athletes groups when seeded from left and right aINS (aINS_L, aINS_R) and dACC seeds, as shown in Fig. 2 and Supplementary Table  S1. There were no differences detected when seeded from the three other seeds, namely the left and right amygdala and vmPFC. Compared to non-athletes, endurance athletes showed significantly increased rsFC in all identified clusters. The differences in rsFC appeared in fourteen clusters, mainly located in autonomic and sensorimotor regions (see Fig. 1 and Table S1). Using aINS_L as seed region, significant rsFC differences were observed in the premotor cortex, posterior insula and CAN areas like the dorsolateral prefrontal cortex (dlPFC), vACC and angular gyrus (AG). Although to a lesser extent, similar clusters showing between group differences in rsFC were found using the right aINS as seed region. In this case, we observed clusters in the left posterior insula and vACC, as shown in Fig. 1 and other cluster in supramarginal gyrus listed in Supplementary Table S1. When seeded from dACC, we found one large cluster in primary sensorimotor cortex (S1/M1) in each hemisphere and other three clusters of smaller size in vACC, AG and premotor areas.

### Network-based statistics

We used the data-driven NBS analysis to detect whole-brain rsFC differences between groups not accounted for by the hypothesis-driven seed-based correlation analysis. This analysis revealed two network components with significantly higher rsFC in endurance athletes (Fig. 2, p < 0.01). The first network components consisted of 13 nodes and 13 edges. Nodes within this network were mainly motor and autonomic regions, distributed across the somatomotor (blue), frontoparietal (orange) and dorsal/ventral attention networks (green and violet). The frontoparietal and ventral attention networks included nodes within the insular and dorsolateral prefrontal cortices. The second network component consisted of 11 nodes and 13 edges, comprising motor-related areas, such as visuomotor and premotor, and autonomic regions, namely AG and vACC, which were mainly distributed across dorsal/ventral attention and frontoparietal networks.

### ALFF/fALFF

We found no differences in ALFF or fALFF between endurance and non-athletes at voxel level p < 0.001. Even without controlling for age and BMI, there were no significant group differences in these measures in any brain region.

### Mediation analysis

**Figure 3:**
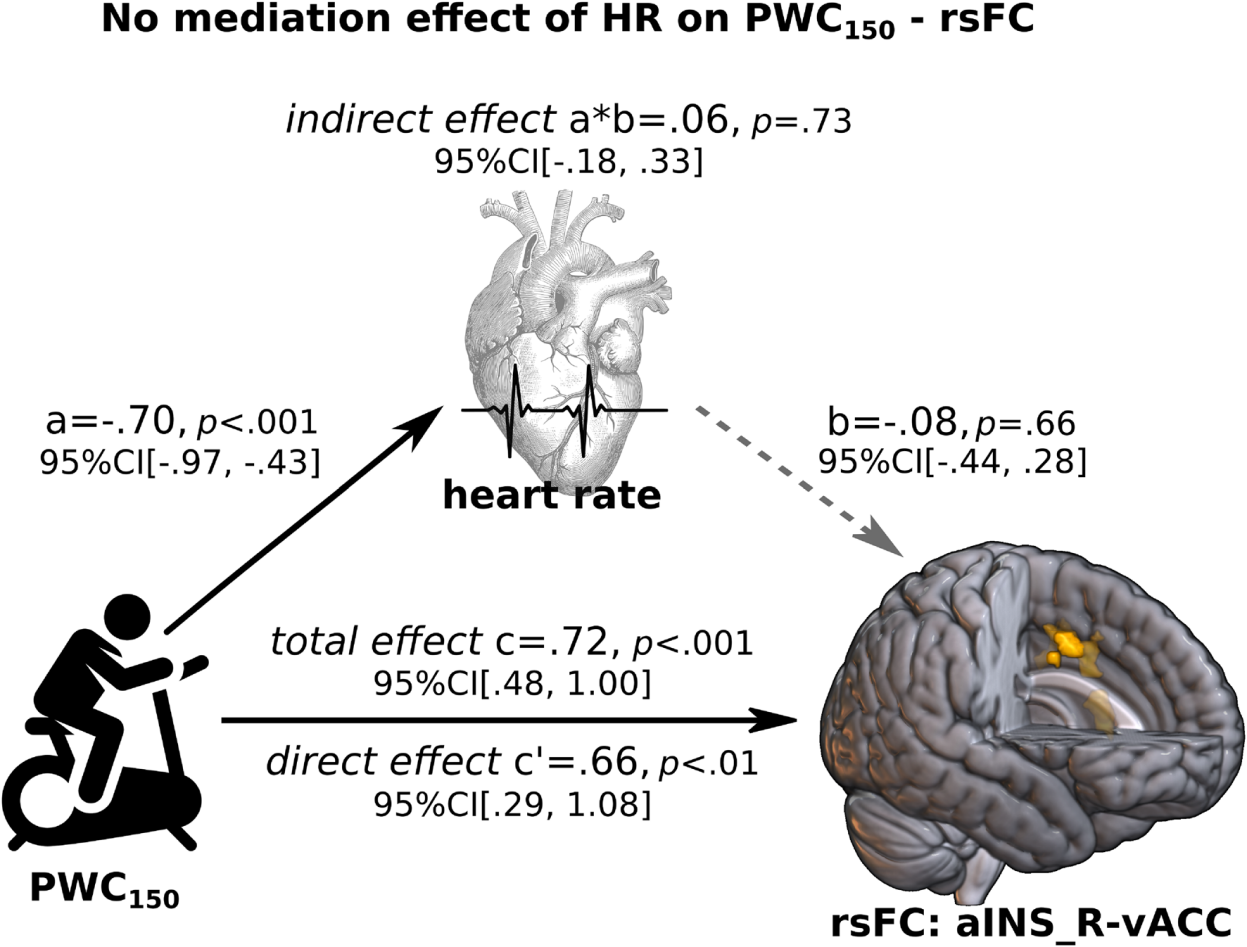
Representative mediation analysis for a given cluster of seed-based rsFC differences (aINS_R-vACC) between athletes and non-athletes adjusted by age and BMI. Alterations in rsFC are due to regular physical exercises and are not mediated by heart rate. Path “a” is the effect of PWC$_{150}$ (causal variable) on heart rate (mediator). Path “b” is the effect of heart rate (mediator) on aINS_R-vACC connectivity (outcome variable), partialling out the effect of PWC$_{150}$. The indirect effect a*b measures the amount of mediation, and the direct effect c’ is the effect of PWC$_{150}$ on rsFC after controlling for heart rate. The total effect is the sum of direct and indirect effects. All path estimates are depicted as standardized regression coefficients with their respective p-value and 95% confidence interval (CI). The dashed gray line indicates the non-significant result obtained for path “b.” Icons for bike, heart and heart rate signal were obtained from freesvg.org. aINS_R - right anterior insula, vACC—ventral anterior cingulate cortex, rsFC—resting-state functional connectivity, PWC$_{150}$—physical working capacity at a heart rate of 150 beats per minute, BMI—body mass index.

We used a mediation analysis across subjects to investigate whether changes in seed-based rsFC were a direct consequence of physical exercises or rather an indirect effect mediated by heart rate reductions. The analysis was conducted on all fourteen clusters exhibiting differences in rsFC between groups (see Supplementary Table S1) with age and BMI as covariates. Results based on 1000 bootstrapped samples indicated that heart rate did not significantly mediate the $\hbox {PWC}_{{150}}$ - rsFC relationship in any cluster. All bootstrap confidence intervals contained 0, indicating that the indirect effect of heart rate was not significant at $\alpha$ < 0.05. Figure 3 shows a representative mediation analysis result for the cluster in ventral anterior cingulate cortex when using the right anterior insula as seed region. We also tested for reversal causal effects by interchanging the mediator and the outcome variable and so have the rsFC causes heart rate. There was also no significant mediation effect of rsFC on the relationship between $\hbox {PWC}_{{150}}$ and heart rate in any cluster.

## Discussion

To investigate the effect of regular endurance training on CAN connectivity and the brain-heart axis, we examined differences in rsFC patterns of six core regions of the CAN between endurance athletes and non-athletes. We used the NBS technique to explore potential whole-brain differences in rsFC not accounted for in the seed-based approach, performed group comparisons of (f)ALFF to investigate regional changes in brain activity, and explored the role of heart rate in mediating the influence of regular physical training on rsFC. Using left and right aINS and dACC as seeds, we found higher rsFC within CAN and sensorimotor areas in endurance athletes. The NBS method agreed with most of the results obtained from the seed-based correlation analysis. We found two network components with higher connectivity in athletes mainly encompassing autonomic regions, including dlPFC, insula, vACC, and AG, and motor-related areas distributed across four major resting-state networks, namely somatomotor, ventral and dorsal attention, and frontoparietal networks. Finally, we demonstrated that heart rate did not significantly mediate the influence of physical exercise on CAN connectivity.

Our findings confirm that intensive physical training might enhance brain connectivity within CAN. Previously, Sie and colleagues^27^ showed enhanced rsFC in autonomic brain areas while investigating patterns of CAN connectivity in baseball players. The rsFC group differences found in the current study mainly occurred within CAN regions and sensorimotor networks, largely mirroring the functional characteristics that distinguish the athlete from the non-athlete brain. Both anterior insulae seeds showed significant rsFC group differences with classical CAN areas in the prefrontal, cingulate cortices or angular gyrus. For example, the presence of clusters in the dlPFC and vACC is not surprising as they are major centers of sympathetic control^26^ and crucial for heart rate regulation. Moreover, a large body of neuroimaging studies suggests that functional changes in dlPFC and vACC occur in response to physical exercise^34,35^. The right anterior insula showed group differences over a greater volume of voxels than its contralateral counterpart in line with the lateralization of these brain regions. Independently of the functional hemispheric asymmetry associated with regular physical training^36^, it has been hypothesized that both anterior insulae might subserve differential functions and are linked to separate circuits. While the right anterior insula communicates with more brain regions and has a specific role in heartbeat awareness and sympathetic activity, the left side is thought to play an essential role in language functions and parasympathetic processing^37,38^.

Interestingly, the NBS analysis yielded a component integrated by regions that are part of the dorsal attention network, e.g. visuomotor (BA 7) and fusiform regions^39^. This result was unexpected as the dorsal attention network is mainly recruited in types of sport requiring fine attentional skills, such as tennis or badminton. Although the function of fusiform and visuomotor areas in the CAN is not entirely established, a recent study revealed that fusiform gyrus appears to be part of a subnetwork specialized in complex autonomic control of the heart^40^. In addition, some of the regions labeled as visuomotor exactly lie within the precuneus cortex which has multiple roles in sympathetic and parasympathetic cardiac control. Thus, the presence of these regions may obey the general functional changes that occur in the CAN of an athlete’s brain.

We observed a negative relationship between heart rate and rsFC of CAN regions, i.e., athletes have slower heart rate and higher rsFC while non-athletes have higher heart rate and lower rsFC. In a recent study of our group^15^, we reported this relationship using different sample cohorts and scanner parameters. Thus, based on this finding and studies on brain-heart interaction^7,8,10,11^, we hypothesized that heart rate mediates the effect of regular physical activity on rsFC. However, our mediation analysis revealed that the brain-heart axis is negligible in physically trained people because the role of heart rate as a mediator was not significant. Yet, it is worth recalling that at rest, the brain-heart interaction is weak and easily confounded by other physiological processes. Indeed, the autonomic outflow has scarcely been investigated at rest but rather via stimuli, which can elicit elevated autonomic responses^41^. The low evoked autonomic response at rest is the reason why most neuroimaging researchers focus on bottom-up investigations of brain-heart interaction in which heart rate fluctuations confound brain functional connectivity and, therefore, treated as physiological noise^19,20^. Following this line of reasoning, we initially used heart rate as the mediator variable between $\hbox {PWC}_{{150}}$ and rsFC in our mediation analysis. The above-mentioned weak influence of heart rate changes on rsFC can explain the non existent mediation effect of heart rate on the relationship between $\hbox {PWC}_{{150}}$ and rsFC. Heart rate fluctuations explain on average less than 15% of the BOLD signal variance^19,42^, and this amount is likely accounted for in path ”a” of the mediation model due to the strong collinearity between $\hbox {PWC}_{{150}}$ and heart rate, which leave no unique variance in heart rate to explain rsFC.

We believe that the combined findings of increased CAN connectivity in athletes and no mediation effect of heart rate on the relation between physical training and rsFC could be specific for the type of sports investigated and the cardiac metric used, i.e. endurance and heart rate. First, we should emphasise that our decision for choosing heart rate is because of its easy interpretability, wide use, and the fact that it indexes both sympathetic and parasympathetic activity. However, other cardiovascular measures may be more appropriate in some instances for assessing the brain-heart axis in athletes. For example, HRV-derived autonomic variables are consistently elevated in elite athletes with ventricle hypertrophy^43^, suggesting that vagal indices may be better markers for quantifying cardiac autonomic changes in these individuals than heart rate. Moreover, we should consider the training load as a key factor for remodeling neural circuitry^2^ and cardiovascular responses^44^. Intermediate levels of training loads are associated with increased vagal drive, whereas intense training volumes shift the cardiovascular autonomic modulation from a parasympathetic toward a sympathetic predominance^44,45^. In addition, exercise-related autonomic changes are also likely to differ across sports modalities. Accumulating evidence indicates that sports with high dynamic demands like endurance promote a physiological profile different to those less physically intense, e.g. golf or billiards^28^.

In contrast to earlier studies, we report no differences in (f)ALFF between athletes and non-athletes. These findings should be carefully interpreted as differences in methodology like physiological noise correction or preprocessing steps order may be the reason for such discrepancies. Another plausible explanation might be the type of sport investigated. It seems that endurance sports cause changes in rsFC by enhancing the coherence of resting-state low-frequency fluctuations between brain regions without regionally altering its amplitude. This is not surprising since (f)ALFF reflects the local properties of specific brain regions, while rsFC reflects the temporal correlation of low-frequency fluctuation between distant brain regions. For example, in a recent study, Zhang and colleagues reported rsFC alterations between the cerebellum and fusiform gyrus in ice-skating athletes not detected by fALFF^46^.

The main limitation of this study is the reduced number of participants. A larger sample size would be particularly important to power the statistical analyses conducted to determine group differences in rsFC and (f)ALFF. The elevated threshold of $\hbox {PWC}_{{150}}$ used as criterion to include endurance athletes also limited the number of available athletes that could participate in the study. Nevertheless, the significant group differences found in rsFC, the use of conservative statistical thresholds and the agreement with a previous study in the field^27^ suggest that our results are reliable. A further limitation was the absence of athletes with different sports experiences. Varying sporting experiences would have helped to investigate whether the level of endurance experience affects CAN connectivity. Moreover, the exclusive presence of male participants raises the question of whether the results are generalizable to female endurance athletes. However, given the known gender differences in functional brain connectivity^47^, including females and males would have made the interpretation of results challenging. Our CAN model does not cover all brain regions putatively involved in heart rate regulation, such as small nuclei located in the brainstem^26^. In particular, we decided not to include brainstem nuclei given the challenges of defining them anatomically and the high sensitivity of the brainstem to physiological and motion artefacts. Furthermore, although we controlled for age and BMI, we cannot exclude the influence of other potential confounders in our results. Finally, we should consider that a longitudinal and not a cross-sectional study is the correct way to test causality between regular exercise training and functional brain changes. In a cross-sectional study as ours, there is always doubt whether functional connectivity changes result from sports adjustments or are instead a precondition for effective and efficient motor performance.

Increased CAN connectivity seems to be a common feature in sportspersons. The present study of seed-based correlation analysis showed that athletes have higher rsFC in autonomic areas like the insula, dACC, vACC, and AG and sensorimotor regions. These results were further confirmed by network-based statistical analysis, where we found two network components with higher connectivity in athletes encompassing autonomic regions and motor-related areas. We report that endurance exercise enhances brain connectivity but does not induce regional changes in brain activity. Overall, our findings provided further evidence that people exercising on a regular basis have increased brain functional connectivity for central autonomic and sensorimotor processing.

## Methods

### Participants

We recruited 20 male endurance athletes and 21 male non-athletes through advertisements posted at the University of Jena, social networks and sports clubs in Jena and its surroundings. This cohort was part of another research project of our group investigating pain-processing in runners and triathletes^48^. The study was approved by the Ethics Committee of the Faculty of Social and Behavioral Sciences of the Friedrich Schiller University Jena in accordance with the ethical guidelines of the current official version (from 2013) of the Helsinki Declaration. All participants provided written informed consent and were compensated for their participation.

Inclusion criteria for all participants were: male (to reduce variability based on gender), age range 18-50 years; no current or past psychiatric, neurological or other medical disease interfering with the investigation. Specific inclusion criteria for endurance athletes were: endurance training for at least 6 hours per week for the last 3 years without any sign of exercise dependence; physical work capacity during heart rate of 150 beats per minute ($\hbox {PWC}_{{150}}$) $\ge$ 3.0 W/kg. PWC is a test to assess the person’s aerobe performance capacity and was performed using an electronically braked bicycle ergometer (Ergometrics 900, Ergoline, Bitz, Germany). Specific inclusion criteria for non-athletes were: no regular participation in any kind of endurance sports; $\hbox {PWC}_{{150}}$ $\ge$ 2.2 W/kg. Three athletes were not included in the current study due to missing physiological data. Demographic data for the final study sample are listed in Table 1.

**Table 1:** Demographic and physiological data. *p* values are given for group comparisons using Mann-Whitney U test.

	Athletes	Non-athletes	*p* value
	(n=17)	(n=21)	
**Biographical data**			
Age (years)	28.8±4.8	26.0±6.1	0.1
BMI (kg/m$^{2}$)	23.0±1.6	24.1±3.1	0.24
**Aerobic fitnes**			
PWC$_{150}$ (W/kg)	3.5±0.5	1.6±0.3	< 0.001
LT (W/kg)	2.7±0.5	1.1±0.2	< 0.001
**Autonomic indices**			
Heart rate (bpm)	54.0±8.7	72.7±9.8	< 0.001
RMSSD (ms)	63.3±27.4	46.1±17.4	< 0.05

BMI—body mass index, PWC$_{150}$—physical work capacity during a heart rate of 150 (watt per kg body mass), LT—lactate threshold (watt per kg body mass), bpm—beats per minute, RMSSD—root mean squared of successive difference (miliseconds).

### rs-fMRI data acquisition

We collected data on a 3T whole body-system equipped with a 12-element head matrix coil (MAGNETOM Prisma, Siemens Healthcare, Erlangen, Germany). Participants were instructed to keep their eyes open during the entire measurement. 1900 whole-brain volume sets were acquired using a multiband multislice GE-EPI sequence (TR = 484 ms, TE =30 ms, flip angle = 90$^\circ$, multiband factor = 8, matrix size = 78 x 78 pixels, voxel size = 2.5 x 2.5 x 2.5 mm3 and with 56 contiguous transverse slices). A high-resolution anatomical T1-weighted volume scan was obtained after fMRI using a magnetization prepared - rapid gradient echo (MP-RAGE) sequence in sagittal orientation (TR = 2300 ms, TE = 3.03 ms, TI = 900 ms, flip angle = 9$^\circ$, matrix size = 256 x 256 pixels, number of sagittal slices = 192, voxel size = 1 x 1 x 1 $\hbox {mm}^{3}$). Heart rate and respiratory activities were recorded during rs-fMRI data acquisition using the scanner’s physiological monitoring system.

### rs-fMRI preprocessing

We used the *afni_proc.py* command in the AFNI software package^49^ to preprocess the rs-fMRI data. The AFNI’s *afni_proc.py* command is a widely used tool to create single-subject processing scripts for fMRI. For reproducibility purposes, we show in the Supplementary Materials the processing steps and options passed to *afni_proc.py* to set up our pipeline. Briefly, after discarding the first twenty volumes, artifacts time-locked to the cardiac and respiratory cycles and slow blood oxygenation level fluctuations were respectively modeled via RETROICOR^50^ and respiration volumes per time (RVT) regressors^51^. Further preprocessing included alignment of each EPI volume to the volume with minimum outlier fraction, spatial registration of the aligned time series data to the anatomical scan, and warping of the anatomical scan to Montreal Neurological Institute (MNI) template. This transformation was also applied to the functional data, which were subsequently smoothed with a 6-mm full-width half-maximum Gaussian kernel. Additionally, we applied a bandpass filter to retain frequencies between 0.01 - 0.1 Hz and reduced contributions of non-neural sources by regressing the following nuisance variables: (1) 12 motion regressors, (2) voxelwise local white matter regressors, and (3) 3 principal components of ventricle signals (ANATICOR^52^). For the generation of white matter and ventricles masks, we used Freesurfer 7.1.0 on the MP-RAGE data (http://surfer.nmr.mgh.harvard.edu).

### Functional connectivity analysis

**Figure 4:**
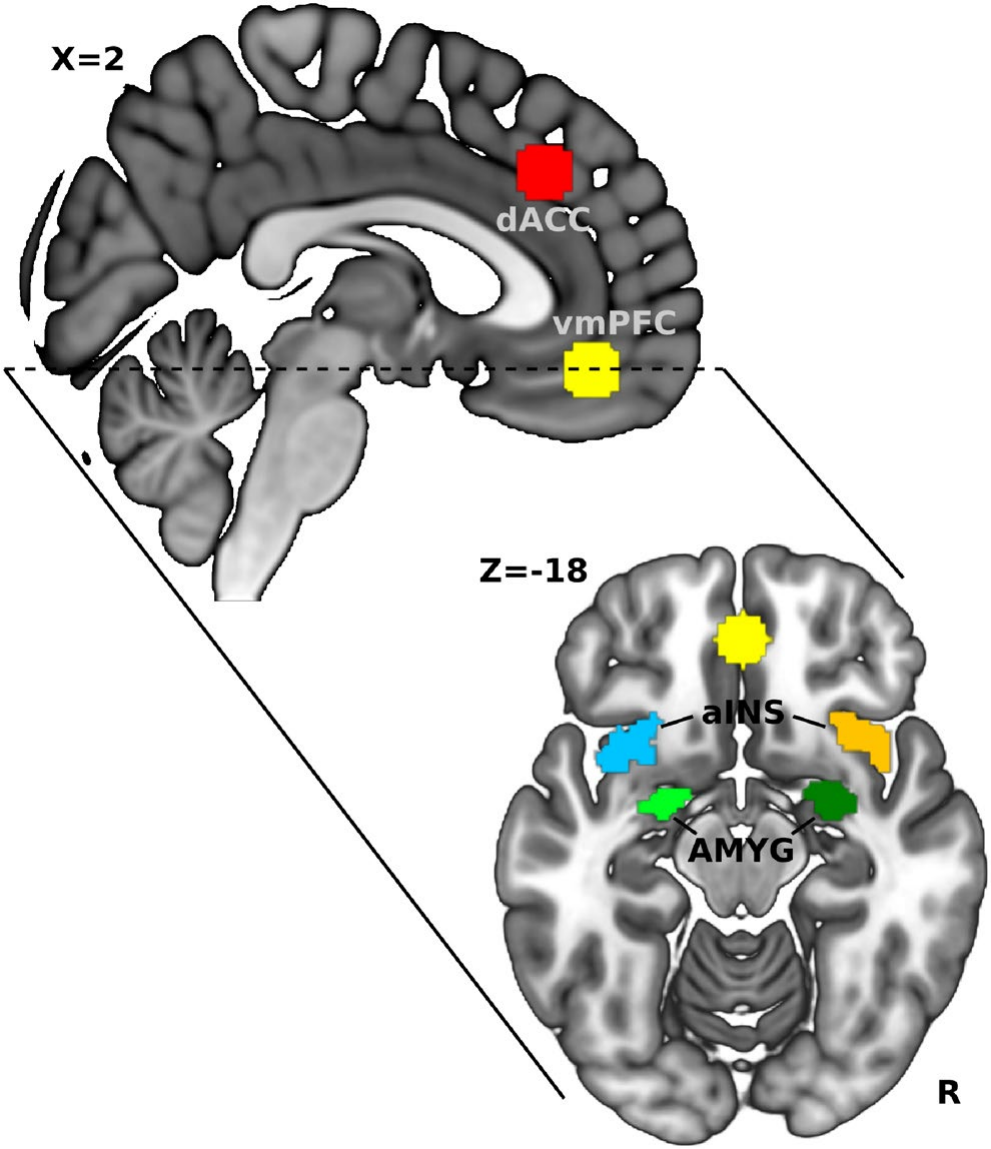
Locations of ROIs used for seed-based rsFC analysis. dACC—dorsal anterior cingulate cortex, vmPFC—ventrolateral prefrontal cortex, aINS—anterior insula, AMYG—amygdala.

We defined six CAN regions-of-interest (ROIs), vmPFC, dACC, and one on each hemisphere of the aINS and amygdala, as seed regions for rsFC. Our previous publication^15^ give details of how ROIs were defined. Briefly, the VMPFC and dACC ROIs were drawn as a sphere of 10 mm radius, respectively centered at MNI-coordinates, x = 0, y = 44, z = 14 and x = 5, y = 32, z = 36. Left and right aINS and amygdala ROIs were created using the Wake Forest University Pick Atlas tool for SPM. Figure 4 shows the locations of all six ROIs.

The average time series of each ROI was correlated via Pearson’s correlation with all brain voxels to generate rsFC maps. These rsFC maps were then transformed to Z maps using Fisher’s Z transformation and compared between groups, controlling for age and body mass index (BMI). The resulting statistical maps were corrected for multiple comparisons using cluster correction with AFNI’s 3dClustSim. This program computes a cluster-size threshold for a given voxelwise p-value threshold, so the probability of anything surviving the dual thresholds is at some $\alpha$. To do this, 3dClustSim uses a Monte Carlo simulation procedure (10000 simulations) that considers the volume size and the level of smoothness associated with the rs-fMRI data. A minimal cluster-size threshold of 59 voxels was necessary for identifying significant differences at $\alpha$ <0.05 with an initial voxelwise threshold of p < 0.001.

### Network-based statistical analysis

In addition to the seed-based rsFC approach, we investigated significant between-group differences in the whole-brain network connectivity using the NBS framework^32^. The main goal of using NBS in our study was to identify potential differences in rsFC not accounted for by the pre-defined seed regions. Individual connectivity matrices were generated by extracting the mean time series from 400 ROIs based on the Schaefer parcellation^53^. With the NBS procedure, we identified components, or networks, using a two-sample t-test at each connection and applying a primary component-forming threshold at t > 5. To assign a statistical significance to each identified component, NBS first permutes n times the group assignments for participants, then identifies components from each permutation using the same t-threshold and generates an empirical null distribution of maximal component sizes. This distribution is then compared against the component sizes identified without permuting the group labels to compute family-wise error (FWE)-corrected p values. We performed 10000 permutations and considered components with p < 0.01 FWE-corrected as statistically significant.

### Amplitude of low-frequency fluctuations

We calculated ALFF and fALFF using the *afni_proc.py* script. These are well-validated, data-driven metrics used to measure the intensity of regional spontaneous neural activity. fALFF is the fraction of ALFF in a given frequency band (here 0.01-0.1 Hz) and is less sensitive to physiological noise than ALFF^33^. fALFF/ALFF differ from rsFC in that they measure spontaneous local brain activity, while rsFC statistically assesses the degree of temporal coherence between spatially separated brain regions. We obtained fALFF/ALFF maps for each subject as part of the standard pipeline described above in the *rs-fMRI preprocessing* section. fALFF/ALFF computation is done after nuisance regression and prior passband filtering steps in the pipeline. To compute both indices, the BOLD time series was first converted to the frequency domain using a Fast Fourier Transform, and the square root of the power spectrum averaged across the entire frequency interval. Finally, fALFF/ALFF were standardized by transforming each individual data to z-scores and then compared across groups, controlling for age and BMI.

### Mediation analysis

Given the relationship between heart and brain function, and the influence of regular physical training on both organs, we performed a mediation analysis across subjects to explore whether seed-based rsFC variations (outcome variable) might be driven by physical exercises (causal variable; $\hbox {PWC}_{{150}}$) through heart rate (mediator). To this end, we used the *mediate* function implemented in the *mediation* R-package. Here, the mediation analysis proceeds in two steps. In the first step, we specified two statistical models: the mediator model for the conditional distribution of the mediator (heart rate) given the causal variable ($\hbox {PWC}_{{150}}$) and a set of the observed covariates (BMI and age) and the outcome model for the conditional distribution of the outcome (rsFC) given the causal variable, mediator and covariates. The validity of the assumptions for linear regression of the two models was ascertained using the gvlma function (*gvlma* R-package). In the second step, we fitted separately the mediator and outcome models and then entered their fitted objects into the mediate function which computes the indirect (amount of mediation of heart rate), direct (effect of $\hbox {PWC}_{{150}}$ on rsFC after controlling for heart rate) and total effects (sum of indirect and direct effects). Considering the small sample size, we estimated confidence intervals for indirect, direct, and total effects using a percentile-based nonparametric bootstrap procedure with 1000 resamples to yield more valid estimates of the above quantities.

## Supplementary Information

Supplementary Table S1.

## Data Availability

The datasets used during the current study available from the corresponding author on reasonable request.
