# Supplementary material for: Central autonomic network alterations in male endurance athletes
Source: Sci Rep. 2022 Oct 6;12:16743. doi: 10.1038/s41598-022-20064-3 (PMC9537279; doi:10.1038/s41598-022-20064-3)
Supplement: Supplementary file 1 — Supplementary Table S1. [file 41598_2022_20064_MOESM1_ESM.pdf]

Central Autonomic Network Alterations in Male Endurance Athletes

Feliberto de la Cruz, Maria Geisler, Andy Schumann, Marco Herbsleb, Zora Kikinis, Thomas Weiss, Karl-Jürgen Bär

Functional Connectivity to:	Left/Right	Broadmann's Area	Cluster Size	Peak (MNI)			Z-value
				X	Y	Z	
seed: left Anterior Insula							
Angular Gyrus	L	39	189	-37	-57	54	4.27
dorsolateral Prefrontal Cortex	R	9	79	50	21	23	4.39
ventral Anterior Cingulate Cortex	L/R	24	77	-8	-2	43	3.94
posterior Insula	R	13	67	40	-17	15	3.93
Premotor Cortex	L	6	60	-45	8	30	4.26
posterior Insula	L	13	59	-38	-2	13	4.44
seed: right Anterior Insula							
ventral Anterior Cingulate Cortex	L/R	24	129	-8	6	35	4.55
posterior Insula	L	13	68	-38	-2	13	4.46
Supramarginal Gyrus	L	40	67	-65	-31	33	3.60
seed: dorsal Anterior Cingulate Cortex							
Primary Sensorimotor Cortex	L	1/4	591	-43	-32	50	4.46
Primary Sensorimotor Cortex	R	1/4	396	42	-29	53	4.47
Angular Gyrus	L	39	145	-50	-57	5	4.41
Premotor Cortex	R	6	81	36	-18	67	3.92
ventral Anterior Cingulate Cortex	L/R	24	66	5	-24	50	4.11

Table S1. Brain regions showing functional connectivity differences between endurance athletes and non-athletes groups. In all regions, endurance athletes showed increased functional connectivity. The significance criterion for detecting clusters was set at $\alpha < 0.05$ determined using AFNI' 3dClustSim (cluster size > 59 voxels, thresholded at voxel level $p < 0.001$).

```
# Options passed to afni_script.py to set up our preprocessing pipeline.
afni_proc.py -scr_overwrite                                                    \
    -subj_id $subj_id                                                          \
    -blocks despike ricor align tlrc volreg blur mask scale regress           \
    -copy_anat $subj_indir/warp/anatSS.WP.nii.gz                               \
    -anat_has_skull no                                                         \
    -anat_follower anat_w_skull anat $subj_indir/warp/anatU.WP.nii.gz         \
    -anat_follower_ROI FSvent epi $subj_indir/fs_ap_latvent.nii.gz            \
    -anat_follower_ROI FSWe   epi $subj_indir/fs_ap_wm.nii.gz                 \
    -anat_follower_erode FSvent FSWe                                           \
    -dsets $subj_indir/bold.nii.gz                                             \
    -tcat_remove_first_trs 20                                                  \
    -ricor_regs $subj_indir/oba.slibase.1D                                     \
    -ricor_regs_nfirst 20                                                      \
    -align_opts_aea -cost lpa                                                  \
    -tlrc_base MNI152_2009_template_SSW.nii.gz                                 \
    -tlrc_NL_warp                                                              \
    -tlrc_NL_warped_dsets $subj_indir/warp/anatQQ.WP.nii.gz                    \
                          $subj_indir/warp/anatQQ.WP.aff12.1D                  \
                          $subj_indir/warp/anatQQ.WP_WARP.nii.gz               \
    -volreg_align_to MIN_OUTLIER                                               \
    -volreg_align_e2a                                                          \
    -volreg_tlrc_warp                                                          \
    -blur_size 6.0                                                             \
    -mask_epi_anat yes                                                         \
    -regress_ROI_PC FSvent 3                                                   \
    -regress_anaticor_fast                                                     \
    -regress_anaticor_label FSWe                                               \
    -regress_bandpass 0.01 0.1                                                 \
    -regress_apply_mot_types demean deriv                                      \
    -regress_run_clustsim no                                                   \
    -remove_preproc_files                                                      \
    -html_review_style pythonic                                                \
    -execute
```
